# Decision space and participation of primary healthcare facility managers in the Ideal Clinic Realisation and Maintenance programme in two South African provinces

**DOI:** 10.1093/heapol/czz166

**Published:** 2019-12-23

**Authors:** Immaculate Sabelile Muthathi, Jonathan Levin, Laetitia C Rispel

**Affiliations:** 1 School of Public Health, Faculty of Health Sciences, University of the Witwatersrand, 27 St Andrews Road, Johannesburg 2193, South Africa; 2 Centre for Health Policy & Department of Science and Innovation/National Research Foundation Research Chair, School of Public Health, Faculty of Health Sciences, University of the Witwatersrand, 27 St Andrews Road, Johannesburg 2193, South Africa

**Keywords:** Decision space, policy implementation, ideal clinic, national health insurance

## Abstract

In South Africa, the introduction of a national health insurance (NHI) system is the most prominent health sector reform planned to achieve universal health coverage in the country. Primary health care (PHC) is the foundation of the proposed NHI system. This study draws on policy implementation theory and Bossert’s notion of decision space to analyse PHC facility managers’ decision space and their participation in the implementation of the Ideal Clinic Realisation and Maintenance (ICRM) programme. We conducted a cross-sectional survey among 127 PHC facility managers in two districts in Gauteng and Mpumalanga provinces. A self-administered questionnaire elicited socio-demographic information, the PHC managers’ participation in the conceptualization and implementation of the ICRM programme, their decision space and an optional open-ended question for further comments. We obtained a 100% response rate. The study found that PHC facility managers reported lack of involvement in the conceptualization of the ICRM programme, high levels of participation in implementation [mean score 5.77 (SD ±0.90), and overall decision space mean score of 2.54 (SD ±0.34)]. However, 17 and 21% of participants reported narrow decision space on the critical areas of the availability of essential medicines and on basic resuscitation equipment respectively. The qualitative data revealed the unintended negative consequences of striving for ‘ideal clinic status’, namely that of creating an illusion of compliance with the ICRM standards. The study findings suggest the need for greater investment in the health workforce, special efforts to involve frontline managers and staff in health reforms, as well as provision of adequate resources, and an enabling practice environment.

Key Messages

- Bossert’s notion of decision space offers the opportunity to assess the degree of choice that primary health care (PHC) facility managers exercise in the implementation of national health policies.
- PHC facility managers reported narrow-to-moderate decision space on the critical areas of the availability of medicines and resuscitation equipment
- Notwithstanding the importance of PHC facility managers to the implementation of health reforms, they were not involved in conceptualizing the Ideal Clinic Realisation and Maintenance programme.
- National health policy implementation could have unintended negative consequences of encouraging a compliance culture when there is insufficient involvement of frontline managers.

## Introduction and background

Health sector reforms are at the forefront of the drive to achieve universal health coverage (UHC; WHO, 2016). Although influenced by country setting and context, the drivers of UHC include demographic and epidemiological transitions, inequalities, increased population demands for access to quality care, technological and scientific developments and the promise of poverty reduction and economic growth (Cotlear *et al.*, 2015). The primary health care (PHC) approach, reiterated in the Astana Declaration, is seen as the foundation of these health sector reforms (WHO, 2018).

In South Africa, notwithstanding significant progress in the 25 years of democracy, inequities in the access to, and quality of, health care remain acute (South African Lancet National Commission, 2019). The fragmentation of health financing between the public and private health sectors is a major cause of these inequities (McIntyre and Ataguba, 2012). Consequently, the introduction of a national health insurance (NHI) system is the most prominent health sector reform planned to achieve UHC in the country (NDoH, 2019a). The NHI proposals, originally contained in a government Green Paper (NDoH, 2011), is a health financing system that is designed to pool funds to provide access to quality, affordable health services for all South Africans irrespective of their socio-economic status (NDoH, 2019a). In concert with global developments, PHC is purported as the foundation of a transformed health system (NDoH, 2019a).

The National Department of Health (NDoH) commenced implementation of the NHI in 11 pilot districts, and in three phases, each phase lasting a period of five years (NDoH, 2017a). The first phase of the NHI programme, which commenced in 2012, did not involve new health financing arrangements, but piloted various interventions aimed at strengthening the PHC level (Genesis, Centre for Health Policy, PWC, 2019). One of these interventions piloted was the Ideal Clinic Realisation and Maintenance (ICRM) programme, a PHC quality improvement programme designed to ‘lay a strong foundation for the implementation of National Health Insurance’ (Hunter *et al.*, 2017, p. 111). The factors that influenced the ICRM programme include the results of the 2011 health facility audit that found sub-optimal infrastructure and quality at PHC facilities, and failure to meet the National Core Standards during the inspections conducted by the Office of Health Standards Compliance (Health Systems Trust, 2012; Fryatt and Hunter, 2014). In light of the planned NHI implementation, the PHC level of the health became a presidential priority for intervention (NDoH, 2014).

In this article, we draw on policy implementation theory (Owens and Bressers, 2013) and Bossert’s notion of decision space (Bossert, 1998) to analyse the implementation of the ICRM programme in PHC facilities in two South African provinces. We bring together three strands at methodological and conceptual levels. First, we use the ICRM programme as a case study of national health sector reforms and their implementation at PHC facility level. Second, we explore the participation of frontline PHC managers in the design of such national reforms. Lastly, we examine the decision space of PHC facility managers regarding the 10 vital (life and death) elements of the ICRM programme, all reportedly under their control (NDoH, 2017b).

We frame the study by providing a brief overview of Bossert’s concept of decision space. We highlight why this concept is both novel and relevant for the study among PHC facility managers. An overview of the ICRM programme, the roles and responsibilities of PHC facility managers, and why their participation is central to the implementation of the ICRM programme follow this overview. We conclude the introduction with a brief summary of previous studies that used the concept of decision space.

### The concept of decision space

Bossert conceptualized the notion of decision space as an approach to analysing the effectiveness of decentralization in developing countries (Bossert, 1998). Decision space is defined as the degree of authority based on formal or informally defined rules, given to local authorities, by a central authority (Bossert, 1998). Formally defined rules are written rules based on the law, informally defined rules are those which local authorities use at their own discretion for decision-making (Bossert, 1998). The framework of decision space identifies the functions of health systems governance, access, human resources, health service organization and/or financing for which local authorities are permitted some degree of authority in decision-making. Bossert’s framework provides three categories of decision space, namely ‘narrow’ (little local choice), ‘moderate’ (a range of choice but limited by central rules) or ‘wide’ (little constraint on local choice) (Bossert, 2016, p. 443).

In this study, we used the horizontal axis of Bossert’s decision space of narrow, moderate, and wide to measure the decision space of PHC facility managers over the ten vital elements of the ICRM programme. These elements focus on PHC facility managers’ functional areas of infection control and prevention, availability of medicine and resuscitation equipment and safe storage of medicines (NDoH, 2017b). The NDoH (the central authority) defines the responsibilities (degree of authority) of PHC facility managers (the delegated ‘authority’) in the ICRM manual (NDoH, 2017b). Hence, in this study, we apply Bossert’s concept of decision space in a novel way to frontline PHC facility managers.

### ICRM programme

An ideal clinic is defined as a clinic with ‘good infrastructure, adequate staff, adequate medicine and supplies, good administrative processes, with sufficient adequate bulk supplies, it uses applicable clinical policies, protocols and guidelines, and it harnesses partner and stakeholder support’ (NDoH, 2016, p. 11). The ICRM programme entails quarterly assessments and grading of PHC facilities, using the quality improvement tools designed by the NDoH (Steinhobel *et al.*, 2015). The assessments are followed by an improvement plan and support to all PHC facilities until they meet the ICRM programme requirements, called ‘ideal clinic status’ (Steinhobel *et al.*, 2015)*.* The assessment tools contain 206 items or criteria, categorized as: 110 important, 68 essential and 10 vital items (NDoH, 2017b). Important items are those that affect the quality of the environment in which healthcare is delivered and essential items are elements that indirectly affect the quality of clinical care rendered to the patients (NDoH, 2017b). Vital items are those requiring immediate and full correction, and if not in place could mean the difference between life and death (NDoH, 2017b). Each PHC facility is required to attain at least 90% of the 10 vital elements in addition to the essential and important items, in order for it be declared an ‘ideal clinic’ (NDoH, 2017b).

### Responsibilities of PHC facility managers

In South Africa, PHC facility managers’ are the persons-in-charge of PHC clinics. These clinics range in size from a one-room, two staff facilities in rural areas, to more than 10 rooms, with a staff complement of around ten in urban areas (NDoH, 2019b). The clinics are open for 8 or 12 hr each day, from Monday to Friday, and provide ambulatory care and a wide range of preventive, promotive and curative services (NDoH, 2019b).

The duties of PHC facility managers include: overall management of the facility, including of financial and human resources (professional nurses, clerks and cleaners); professional development of staff, the provision of holistic comprehensive ambulatory care to patients; administrative duties, including the collection of routine information; and quality assurance and compliance with PHC standards (NDoH, 2009; Gauteng Province Department of Health, 2019).

In this study, we focus on PHC facility managers’ functions regarding the vital elements, which cover the following four aspects: infection control and prevention, safe storage of medicines, availability of essential medicines and availability of medical equipment (NDoH, 2017b). The responsibilities of the facility managers in relation to these four components are shown in Table 1.

**Table 1:** Responsibilities of PHC facility managers on four vital elements

Vital category	Brief description
Infection control and prevention	Ensure safe disposal of sharps by ordering enough stock of impenetrable, tamperproof containers for disposal of sharps Train staff on national policy of infection control and prevention Delegate a person to observe these tasks on day to day basis
Availability and safe storage of good quality essential medicines	Monitor stock availability Determine the re-order levels of medical and surgical supplies Order replenishment to maintain minimum and maximum stock Obtain approval from sub-district managers for submission to the district pharmacy If the order is not delivered on scheduled time, follow up with district pharmacy Ensure all medicines are stored at safe temperature Delegate personnel daily to attend to this deliverable
Ensure availability of medical equipment	Monitor availability of essential equipment and required furniture Ensure availability of resuscitation equipment on emergency trolley Replenish emergency trolley equipment daily (where relevant) Order the equipment using the national standardized catalogue for equipment Assign a professional nurse to ensure on a daily basis that the emergency equipment are available, clean and functional

*Source:* Adapted from ICRM Manual (NDoH, 2017b).

The procedures for ordering of medical and non-medical supplies, supply chain management delegations, the availability of support for facility managers to procure and on spending delegations at facility level vary greatly across the country (NDoH, 2014). In general, PHC facility managers have to follow multiple procedures to place their orders. On average approval procedures from PHC facility to district level could take 39 days provided that the items cost less than R500 000 (US$33 300, 1US$=R15), as this is the delegation at district level in most provinces (NDoH, 2014). In most provinces, supply chain management delegations rest with the district, with none at sub-district level. Some PHC facilities are allowed discretionary spending for items less than R2 000 (US$133), but they rely mostly on supply chain management support at district level (NDoH, 2014).

The managerial function of equipment ordering requires the availability of a facility budget. According to the ICRM programme manual, each district should ensure a dedicated PHC facility budget, and involvement of facility managers in budget discussions at district level, to enable managers to influence the facility’s budget allocation (NDoH, 2017b). The district should allocate financial resources in line with the needs of the individual PHC facility (NDoH, 2017b). In turn, the facility managers should develop control measures for rational budget utilization and expenditure and query any financial discrepancies with the district (NDoH, 2017b).

Hence, the PHC facility managers are the main implementers of the ICRM programme, with support from the district, provincial and national departments of health (NDoH, 2017b). Scholars have suggested that the PHC facility managers hold one of the most important positions in the health system, because they have to interpret national policies and steer implementation of health reforms at PHC level, for the benefit of patients, communities and staff (Wilson *et al.*, 2014; NDoH, 2015; Munyewende *et al.*, 2016). However, a precondition for the successful implementation of national health policies and/or reforms is their involvement and participation in such initiatives, and the degree of choice or decision space that they exercise in the implementation of national health policies or reforms.

We defined participation as taking part in ICRM conceptualisation (i.e. the idea and design) (Baporikar, 2014) and implementation (putting into practice the plan) of the ICRM programme. This is because policy implementers are ‘street level bureaucrats’, whose capacity, knowledge, interpretation, motives, power and discretion influence the successful implementation of the policy or achievement of policy goals (Lipsky, 2010; Owens and Bressers, 2013; Juma *et al.*, 2014; Kunaviktikul, 2014). This successful implementation in turn is influenced by the involvement of implementers earlier on in the conceptualization of the policy (Juma *et al.*, 2014), in this case the ICRM programme.

### Studies on decision space

The decision space approach has been applied in numerous studies in low- and middle-income country settings, focusing on health systems governance, access, human resources, health service organization and/or financing (Bossert and Mitchell, 2011; Mohammed *et al.*, 2015; Marchildon and Bossert, 2018). The majority of these studies have found the centralization of human resource and financial management decisions, with consequent narrow decision space of district health managers (Mohammed *et al.*, 2015; Kigume *et al.*, 2018; Rispel and Moorman, 2018; Sumah and Baatiema, 2018). In contrast, Alonso-Garbayo examined district health managers’ use of decision space in human resource management in Uganda, and found that these managers made decisions beyond their legal authority, hence exercised wide decision space (Alonso-Garbayo *et al.*, 2017).

Several studies have focused on the factors that influence decision space, with consensus that capacity building is critical to improving the exercise of decision space (Bossert and Mitchell, 2011; Bossert *et al.*, 2015; Liwanag and Wyss, 2019). In Tanzania, Kigume and Maluka (2018) explored the influence of institutional capacities in decision space, and found that limited capacity in financial resources affected both the ability and exercise of decision space. Roman *et al.* (2017) found that the availability of resources, management capacities and accountability mechanisms influence the exercise of decision space positively.

In South Africa, healthcare system remains centralized, with narrow decision space over finance and human resources at district level (Rispel and Moorman, 2018). District managers have limited influence over allocated budget and no control over human resource planning and appointments (Wolvaardt *et al.*, 2013).

Notwithstanding the substantial body of literature on decision space in the context of health sector decentralization, there is dearth of studies on the decision space of frontline health facility managers. Since PHC facility managers are central to the successful implementation of the ICRM programme, this study aimed to explore their degree of authority (decision space) and their participation in conceptualizing and implementing the ICRM programme.

## Methods

### Application of Bossert’s decision space concept

We used the horizontal axis of Bossert’s decision space framework to measure decision space of PHC facility managers on the vital elements of the ICRM programme that are included in their responsibilities. We defined narrow decision space as little control, moderate as partial control and wide as full control (Table 2).

**Table 2:** Application of Bossert’s framework

Functional areas of PHC facility managers	10 Vital elements of the ICRM programme indicator	Narrow (little control)	Moderate (partial control)	Wide (full control)
Infection prevention and control	Sharps containers are disposed when they reach limit mark			
	Sharps are disposed in impenetrable, tamperproof containers			
	There is one functional wall mounted room thermometer in the medicine room			
Ensuring availability and safe storage of medicine	Medicine room temperature is recorded daily			
	Medicine room temperature is kept within safe limits			
	Cold chain procedure for vaccines is maintained			
	90% of medicines on tracer medicine list is available			
Ensuring availability of medical equipment	Functional basic resuscitation equipment is available			
	Emergency trolley is restored daily or after use			
	Oxygen cylinder with a pressure gauge is available			

*Source:* Adapted from ICRM Manual (NDoH, 2017b) and Bossert’s framework (Bossert, 1998).

### Study setting

We were interested in the implementation of the ICRM programme in the NHI pilot sites. Hence, the study setting was the two NHI pilot districts located in Gauteng province (GP) and Mpumalanga province (MP) of South Africa. The criteria for the selection of these two provinces were: NHI pilot district, geographical proximity, budgetary and logistical considerations, including geographical variations, as GP is an urban province, whereas MP is predominantly rural (Statistics South Africa, 2018). In GP, the NHI pilot district was the City of Tshwane, and in MP, it was Gert Sibande district. Table 3 compares key features of the two districts.

**Table 3:** Description of the selected districts

	City of Tshwane district	Gert Sibande district
Population size	±3, 3 million residents	±1, 1 million residents
Healthcare centres	64 day clinics, 10 Community health centres	63 day clinics, 15 community health centres
Compliance to ICRM programme	89% of PHC facilities complied with the requirements in 2017	72% of PHC facilities complied with requirements in 2017
Reasons for failure to comply	Shortage of essential medical equipment, lack of cleaning material, shortage of staff	Shortage of essential medical equipment, lack of cleaning material, shortage of staff and poor infrastructure
Supply chain management delegations	Supply chain managed at district office	Supply chain managed at district office and at hospitals which are linked to PHC facilities per sub-district

*Sources:* Massyn *et al*.(2017), Statistics South Africa (2018), Mpumalanga Department of Health (2017) and Gauteng Department of Health (2017) .

### Study design

We conducted a cross-sectional survey among PHC facility managers to analyse their decision space and participation in the ICRM programme.

### Study population and sampling

The population of interest was all the PHC facility managers in-charge of day clinics (*n* = 127; GP = 64; MP = 63), defined as those PHC facilities that were open for 8 or 12 hr each day and that were part of the ICRM programme. All 127 PHC facility managers were invited to participate in the study.

PHC facility managers in-charge of community health centres, mobile and satellite clinics were excluded from the study, because of the variation in the community health centres, the differences in resource allocation and functioning between clinics and community health centres and the differences in service delivery and populations served. The ICRM programme is not implemented in mobile and satellite clinics.

### Data collection instrument

We developed a self-administered questionnaire (SAQ) to evaluate PHC facility managers’ participation in the conceptualization and implementation phases of the ICRM programme, as well as their decision space on the 10 vital elements of the ICRM programme.

The questionnaire consisted of four sections: 11 questions on background and demographic characteristics, 9 questions on participation in the conceptualization and implementation of the ICRM programme and 10 questions on decision space in the ideal clinic implementation. The background and demographic characteristic section collected information on age, gender, qualifications, years of experience and years of involvement in training. The section on participation consisted of a series of statements on participation in planning, leadership, negotiation for resources and sharing of knowledge about the ICRM programme. A 7-point Likert scale was used to measure PHC managers’ participation, where 1 indicated strongly disagree and 7 indicated strongly agree. The section on PHC managers’ decision space contained ten statements derived from the ten vital elements of the ICRM programme manual that are under the control of the PHC facility managers. These 10 statements were measured using a 3-point scale based on Bossert’s framework for mapping decision space, namely 1 (narrow), 2 (moderate) and 3 (wide) (Bossert, 1998). The final section consisted of an optional open-ended question to allow PHC facility managers to comment on any aspect related to their participation or decisionspace related to the ICRM programme.

Prior to implementation, we pre-tested the tool with three PHC facility managers from outside the study districts and no adjustments were necessary. These responses were excluded from the main study.

### Data collection

The completed questionnaire was uploaded into Research Electronic Data Capture (REDCap) a secure, web-based application designed to support data capture for research studies (Harris *et al.*, 2009) and hosted at the author’s institute. We conducted the study between October 2017 and February 2018. We contacted the PHC facility managers to enlist their participation and to plan the date for fieldwork. On the survey day, we held an introductory meeting with each of the PHC facility managers to explain the study and to request their voluntary participation.

Following informed consent, each facility manager completed the SAQ on a tablet, and the principal researcher assisted if necessary. The tablet allowed for text input by the facility manager or digital recording of additional comments, which were optional.

### Data management and analysis

#### Quantitative data

The quantitative data were extracted from REDCap and imported into STATA^®^ 15 for analysis. We used descriptive statistics to analyse the socio-demographic data.

#### Participation

We analysed the participation of the PHC facility managers in conceptualizing and implementing of the ICRM programme. We calculated frequencies and percentages for each of the seven items in the participation category. The overall mean score for participation was calculated by averaging the participants’ mean scores for all the items within the participation category.

#### Decision space

We analysed decision space of PHC facility managers on the 10 vital elements of the ICRM programme. The frequency distribution of decision space scores on narrow, moderate and wide was computed. The overall mean score for decision space was computed by averaging the participants’ mean scores for all the items within the decision space category.

Using a one-way ANOVA, a comparison of overall mean score for decision space by socio-demographic characteristics was done, to determine which background demographic characteristics were associated with decision space in the implementation of the ICRM programme. After initially including all demographic characteristics in the model, we did an exploratory analysis to determine which variables were the best predictors of decision space and participation using a backward elimination algorithm (Vittinghoff *et al.*, 2012). All tests were conducted at 5% significant levels.

#### Qualitative data

Responses to the open-ended question were either transcribed verbatim, or exported from the REDCap file into a word document. All typed comments corresponded to the unique identifiers used for the survey.

The qualitative comments were analysed using thematic analysis (Maguire and Delahunt, 2017). Three researchers (including the supervisor) analysed the raw data of eight participants separately. We read and reread transcripts to familiarize ourselves with the data and to get a sense of the whole. We made notes of our earlier impression next to the text. We coded line by line through underlining each text segment that indicated participants’ views, we used terms emerging from the data to label these text segments forming codes. After this phase, we had a coding meeting to discuss and agree on all the codes. The codes were grouped into common themes. Following agreement on codes, the principal researcher analysed the remaining interviews.

### Reliability and validity

#### Quantitative data

During the data collection tool design phase, the questionnaire was distributed to a team of researchers, who reviewed it for face validity (Heale and Twycross, 2015). Pilot testing the tool also allowed for testing questions if they were understood by the participants. We calculated the Cronbach’s alpha to establish internal consistency of all the items in each subscale of the questionnaire. Participation and decision space categories had Cronbach’s alpha 0.77 and 0.81, respectively, which indicate good internal consistency (Tavakol and Dennick, 2011).

#### Qualitative data

We applied Lincoln and Guba’s criteria of trustworthiness to examine rigour in this study (Nowell *et al.*, 2017). The principal researcher listened repetitively to the audio-recorded comments to ensure credibility of the findings and read and reread the written comments ensuring prolonged engagement with the data. Two other researchers coded the data, and inter-coder agreement was reached. Attaching the extracts of narratives in report writing to illustrate themes ensures confirmability. To ensure dependability, all the raw data and the coded data are archived to illustrate how we reached theme agreement.

### Ethical considerations

The Human Research Ethics Committee (Medical) of the University of the Witwatersrand provided ethical approval for the study. We also obtained study approval from the provincial and local government health departments. We gave all participants a detailed information sheet, as well as a verbal explanation of the study, which included the voluntary nature of participation, confidentiality and anonymity.

All study participants provided written consent, provided written consent, via Research Electronic Data Capture, a secure, web-based application designed to support data capture for research studies. All data collection forms had unique identifiers, and the results of the study are presented in aggregate form.

## Results

All PHC facility managers in GP and MP provinces agreed to participate in the study (*n* = 127), hence a survey response rate of 100% was obtained (GP = 63, MP = 64). We begin by presenting the quantitative results, followed by the presentation of qualitative findings.

### Quantitative results

#### Socio-demographic characteristics

The majority of participants (90.55%, *n* = 115) were female and their mean age in years was 51 (SD ±7.2). Most participants (86.61%, *n* = 110) had received training on the ideal clinic, although almost half of the trained participants (43.64%, *n* = 48) only received one day of training (Table 4).

**Table 4:** Socio-demographic characteristics

Characteristics	GP, *n* = 63	MP, *n* = 64	Total, *n* = 127
Gender			
Female, *n* (%)	59 (93.65)	56 (87.50)	115 (90.55)
Male, *n* (%)	4 (6.35)	8 (12.50	12 (9.45)
Age			
Mean age (SD)	53 (7.03)	49 (6.90)	51 (7.19)
Experience			
Mean number of years as PN (SD)	28 (7.85)	22 (8.68)	25 (8.77)
Heard of NHI, *n* (%)	63 (100.0)	63 (98.44)	126 (99.21)
Permanent position, *n* (%)	59 (93.65)	57 (89.06)	116 (91.30)
Training			
Received training on ideal clinic, *n* (%)	51 (80.95)	59 (92.19)	110 (86.61)
Received one day training, *n* (%)	37 (72.55)	11 (18.64)	48 (43.64)
Received week long training, *n* (%)	6 (11.76)	24 (40.68)	30 (27.27)
Received other training, *n* (%)	8 (15.69)	24 (40.68)	32 (29.09)

#### Participation in conceptualization and implementation

**Figure 1:**
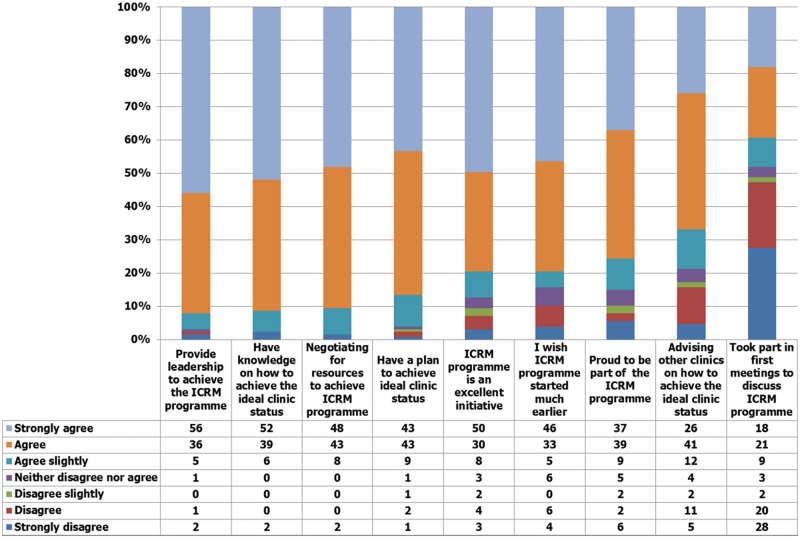
Frequency scores on items within the participation category

An overall participation mean score of 5.77 (SD ±0.90) was obtained regarding participation in the ICRM programme. Only 37% of participants (*n* = 47) agreed strongly that they were proud to be part of the ICRM programme. Less than half of the participants (48%, *n* = 61) agreed to have been part of the very first meetings to discuss the ICRM programme, and 56% (*n* = 71) agreed strongly that they provide leadership in the ICRM programme (Figure 1).

#### Decision space in ICRM programme

Table 5 shows the frequency distribution of scores on decision space items. The overall decision space mean score was 2.54 (SD ±0.34). Although participants reported moderate-to-wide decision space for the majority of the vital items of the ICRM programme, 17 and 21% of participants reported narrow decision space on ensuring the availability of tracer medicines and basic resuscitation equipment, respectively.

**Table 5:** Frequency scores on items within decision space category

Vital elements	Narrow, *n* (%)	Moderate, *n* (%)	Wide, *n* (%)
Sharps containers are disposed when they reach limit mark	7 (5.51)	37 (29.13)	83 (65.35)
Sharps are disposed in impenetrable, tamperproof containers	6 (4.72)	33 (25.98)	88 (69.29)
There is one functional wall mounted room thermometer in the medicine room	10 (7.87)	54 (42.52)	63 (49.61)
Medicine room temperature is recorded daily	1 (0.79)	17 (13.39)	109 (85.83)
Medicine room temperature is kept within safe limits	5 (3.94)	37 (29.13)	85 (66.93)
Cold chain procedure for vaccines is maintained	3 (2.36)	27 (21.26)	97 (76.38)
90% of medicines on tracer medicine list is available	21 (16.54)	64 (50.39)	42 (33.07)
Functional basic resuscitation equipment is available	27 (21.26)	66 (51.97)	34 (26.77)
Emergency trolley is restored daily or after use	3 (2.36)	31 (24.41)	93 (73.23)
Oxygen cylinder with a pressure gauge is available	2 (1.57)	45 (35.43)	80 (62.99)
The overall decision space mean score			$\bar{x}=2,\;54,\;SD=0.34,$

#### Predictors of decision space

Table 6 shows the results for the multiple regression analysis to determine the predictors of decision space. Managing a facility of choice and training of at least 1 week were predictors of PHC managers’ decision space (*P* = 0.001).

**Table 6:** Factors that influence decision space

Multiple regression analysis				
Variable	Level	Coeff.	95% CI	*P*-value
Managing a facility of your choice	No	0 (baseline)		
	Yes	0.30	0.12; 0.49	0.001
Type of training received	1-day workshop	0 (baseline)		
	Week long training	0.18	0.03; 0.33	
	Other trainings	0.26	0.12; 0.41	0.001

#### Qualitative results

The majority of participants (115/127 = 91%) chose to make additional comments. Although these overlap, three main themes emerged: lack of involvement, lack of control and creating an illusion of compliance (Table 7).

**Table 7:** Emerging themes and subthemes

Themes	Subthemes
Lack of involvement	Top down policy-making
	Apparent ongoing exclusion of frontline managers in policy revisions
Lack of control	Budget
	Supply chain management
	Penalties on areas beyond facility manager’s control
Creating illusion of compliance	Self-funding for clinic needs Borrowing of equipment

#### Lack of involvement

Most participants were of the opinion that policy-makers planned the ICRM programme without their involvement. They expressed concern about their lack of involvement in the conceptualization and the planning of the ICRM programme and pointed to their exclusion from providing inputs into the ICRM policy document.

*Most of us as facility managers were not involved when the ideal clinic was conceptualised; it came to our attention as a government policy, which had to be, implemented* (Participant 8, GP).

*We were not involved at the planning as a result there has been number of versions, they keep changing because they [NDoH] have no understanding of how feasible are the planned versions* (Participant 39, GP).

*During the planning phase I was neither involved in providing inputs nor in decision making. The ideal clinic initiative (ICRM programme) was just brought to us as it is* (Participant 24, MP).

#### Lack of control

The PHC clinic managers felt that they lacked control over the facility budget, staff and supply chain management. Some reported that they were penalized for areas beyond their control.

The PHC clinic managers lamented about the very limited budget, and that they were not allowed to shift funds within the allotted budget. They also did not have petty cash. Some PHC facility managers were of the opinion that there was a lack of transparency in the budget especially when the PHC facility budget was managed at hospital level.

*We get limited budget, and we have no control over budget and there is no shifting of funds when they are allocated to certain items. We have no allocated petty cash* (Participant 31, MP).

Most participants were of the opinion that they had no powers to influence procurement processes and turnaround times and that their decision-making powers for the provision of equipment for vital elements were limited since the delivery of orders depended on the supply chain department. Some reported problems in relation to supply chain management were delays, delivery of wrong orders after a very long waiting period and lack of feedback on the progress of procurement. These delays led to managers on some occasions to buy items from their own pocket.

*To achieve the requirements of the [ICRM] programme we rely on supply chain management. We don't have any power to purchase; we keep on doing request after request. That's the only thing that is killing us. And they [supply chain department] don't come back to you to say you will not get this because of this reason. You will see [the reason] after a year when they return the request form* (Participant 45, GP).

*You order equipment, but it is not delivered on time, or the equipment required in terms of the ideal clinic, it's not the one delivered* (Participant 25, GP).

Most participants were of the opinion that they were not equipped with the necessary resources by the national or relevant provincial department of health. Some participants were of the opinion that there was great urgency for the facilities to comply with the requirements of the ICRM programme but less urgency for those in authority to deliver the resources to support the programme. This constrained facility managers’ effectiveness in their functional areas. PHC facility managers highlighted times when they were penalized for areas beyond their control and were expected to deliver without resources from national and/or provincial level.

*There are lots of policies that are required and we are penalised for and we are not responsible for making them available, like the policy on referral, or the memorandum of understanding (MOU) between Department of Health and South African Police Services. MOUs are supposed to be done by Department of Health. There are lots of things that at our ground level we don't have and it is not our fault, but that of those at higher levels* (Participant 25, MP).

#### Creating an illusion of compliance

Most respondents were of the opinion that achieving ‘ideal clinic status’ was strenuous, frustrating and a burden. The pressure and urgency to be compliant led at times to ‘faked’ compliance. The majority of PHC facility managers reported that on some occasions they had no option but to buy out of pocket or borrow equipment required for the ICRM programme, or to improvise to have the required item. Some participants who had improvised using laminated material to provide for signage in the PHC facility, verbalized that they regretted the improvisation as this led to lack of proper signage in the facility by the responsible department.

*When you order the items are out of stock, or they give you two items that are about to expire, when they expire you have to re-order, or have to borrow and paint a good picture that things are fine during assessments, when assessment passes, you have to take those borrowed items back* (Participant 18, GP).

*We don't have medical equipment; sometimes you have to buy from your own personal funds* (Participant 56, MP).

## Discussion

This is one of the first studies in South Africa that used the notion of decision space to assess the degree of choice that PHC facility managers exercise in the implementation of national health policies. The study found that PHC facility managers reported lack of involvement in the conceptualization of the ICRM programme, high levels of participation in implementation and narrow-to-moderate decision space on the critical areas of essential medicines and resuscitation equipment. The qualitative data supported the survey data on these managers’ lack of involvement, as well as the unintended negative consequences of striving for ‘ideal clinic status’, namely that of creating the illusion of compliance with the ICRM standards.

There is evidence that the involvement of frontline staff in policy development or health reforms increases ownership, co-responsibility and successful policy implementation (Veronesi and Keasey, 2009; Lipsky, 2010; Kickbusch and Behrendt, 2013; Levin *et al.*, 2016; Brooke-Sumner *et al.*, 2019). In contrast, excluding frontline staff or managers could lead to role confusion, limited capacity and lack of or unsuccessful policy implementation (Kunaviktikul *et al.*, 2010; Kamanga, 2018). Notwithstanding the importance of PHC facility managers to the implementation of health reforms in South Africa, fewer than half (48%) reported that they took part in the conceptualization of the ICRM programme (Figure 1). Their lack of involvement in ICRM conceptualization also emerged as a theme from the qualitative data. Studies in South Africa and in other countries, albeit with different research questions, have also found the lack of frontline managers in policy conceptualization. In Burundi, the policy decision on the abolition of user fees was made without involving the frontline managers, who felt that the policy objectives were neither clear nor covering those who needed to benefit (Conway and Monks, 2011). In Ireland, frontline managers also felt that they were not consulted on key healthcare reforms (Nimpagaritse and Bertone, 2011). A 2014 South African study that examined management dynamics at PHC clinics in Gauteng and Free State provinces found that only one-third of PHC nursing managers were aware of the NHI (Munyewende *et al.*, 2014). Ditlopo *et al.* (2014) found that nurses’ participation in policy-making is both contested and complex, with differences in the awareness of policies examined between nursing leadership and frontline nurses. Scott *et al.* (2012) also reported exclusion of frontline managers on decisions regarding an equity-promoting staff allocation policy in South Africa, and that such exclusion resulted into low staff morale.

In our study, the majority of PHC facility managers reported high levels of agreement on the questions that focused on participation in the implementation of the ICRM programme. This could reflect social desirability bias, because they had no choice in implementing the programme, as it is part of their responsibility as PHC clinic managers.

Bossert’s approach on decision space offered the opportunity to assess the degree of authority that these PHC facility managers in the two provinces exercised in the implementation of the ICRM programme. Although they reported moderate-to-wide decision space for the majority of the vital elements of the ICRM criteria, 17% of them indicated that they had narrow decision space on the availability of essential medicines while 21% of participants indicated that they had narrow decision space on the availability of resuscitation equipment. In response to the open-ended question, many of these PHC facility managers voiced their frustrations over the lack of control over the budget and supply chain management, which constrained their ability to ensure the availability of medicines and basic resuscitation equipment in these facilities.

Another study that focused on PHC nursing managers in South Africa found that centralized budgets and shortages of medicines in PHC facilities influenced the practice environment negatively, which in turn distressed these managers greatly (Munyewende and Rispel, 2014). A 2015 survey that estimated the extent of stock-outs of antiretroviral and tuberculosis (TB) medicines in public health facilities across South Africa found that 20% of the surveyed facilities reported a stock-out of at least one antiretroviral and or TB-related medicine on the day of contact and 36% during the three months prior to contact (Hwang *et al.*, 2019). Essential medicines and medical equipment are critical building blocks of a health system (WHO, 2010). The absence of essential medicines and medical equipment leads to distrust of the healthcare system by the public, decreases utilization of a healthcare facility by the public (Moyimane *et al.*, 2017) and could even lead to death (Walker *et al.*, 2017). In another study conducted in the Western Cape province of South Africa, albeit with different research objectives, PHC facility managers stressed that being expected to do more with less resources constrained their adoption of new innovations (Brooke-Sumner *et al.*, 2019).

Capacity building improves the ability to exercise decision space, (Liwanag and Wyss, 2019). In our study, at least 1 week of training was a significant predictor of decision space, but training alone is an inadequate mechanism to capacitate implementers without the provision of adequate resources. We could not find other studies that used Bossert’s decision space approach at PHC facility level. Nonetheless, other studies that have examined decentralization have found that the availability of resources and managers’ control over these resources positively influence their ability and extent of exercising available decision space (Marchildon and Bossert, 2018; Roman, 2018).

The PHC managers reported that the pressure, whether real or perceived, to meet the ‘ideal clinic status’, trumped their lack of authority and control and led to unintended consequences of creating an illusion of compliance. They ended up borrowing and buying items from their pocket in order to meet the ICRM programme criteria. Bossert (2014) notes that implementing authorities often bend the rules when the original rules are not enforced, and Lipsky (2010) states that implementers use their discretion in what or how to implement policies.

However, the consequences of acting as compliant could challenge the sustainability of the ICRM programme. Hodes *et al.* (2017) investigated the coping strategies of PHC facility managers with drug stock-outs in the Eastern Cape province of South Africa and found that they formed networks of borrowing drugs from each other’s facility within a district. This practice obscured the severity of their medicine stock-outs and made it difficult to measure the extent of the problem.

The cross-sectional nature of the study means that it represents the perceptions of PHC managers at a point in time. The PHC facility managers are both frontline staff who implements national policy, while at the same time policy actors, who shape the outcome of national health policy. Hence, the results may reflect some social desirability bias. The notion of decision space is uncommon among frontline staff, and may have been misunderstood by these PHC facility managers; hence the high mean scores on overall decision space. The study was done in the two NHI pilot districts in two South African provinces, and the results may not reflect the situation in the other districts in the two provinces and/or in the rest of South Africa.

However, the study has numerous strengths. We obtained a 100% response rate among study participants. Although the exclusion of frontline managers in the conceptualization of health policy is not new, the study has generated new knowledge on the implementation of the ICRM programme in two South African provinces, specifically their decision space and the unintended negative consequences of a compliance culture that obscures health system deficiencies. This is important because the ICRM programme is a major health policy initiative under the auspices of the phased NHI system (NDoH, 2017a), South Africa’s major reform towards UHC. The methodological approach is novel, as it is one of the first studies in South Africa to use Bossert’s decision space approach at PHC facility level. The survey combined the opportunity to elicit open-ended responses, which revealed the contradictions and nuances of policy implementation from the perspective of frontline facility managers.

In South Africa, all PHC facility managers are professional nurses, as also shown in this study. In the world innovation summit for health report (2018), WHO emphasize that ‘nurses and midwives are at the heart of progress towardsUHC and the Sustainable Development Goals (SDGs), because they play a critical role in transforming health policies’ (Crisp *et al.*, 2018, p. 5).

South Africa’ s preparations for NHI system are underway; hence, the findings can inform future health sector reforms. First, the findings imply that policy-makers and executive managers at national and provincial levels should ensure greater investment in the health workforce (Crisp *et al.*, 2018). Second, there should be special efforts to involve frontline managers and staff in discussion on future health reforms, both at their workplaces, as well as through their representative organizations. This should go beyond merely informing them, to active involvement in shaping policies or reforms, as buy-in is likely to achieve successful implementation and reduce unintended negative consequences (Nimpagaritse and Bertone, 2011). Lastly, the findings suggest the need to ensure adequate resources, an enabling practice environment, and support to PHC facility managers, within an overall context of prioritization of the PHC approach as reiterated at Astana (WHO, 2018).

## Conclusion

The study has generated new knowledge on the degree of choice that PHC facility managers exercise in the implementation of the ICRM programme in South Africa, and the potential for unintended negative consequences of national health policy implementation when there is insufficient involvement of frontline managers. PHC is purported as the central plank of NHI reforms in South Africa. Hence, the success of PHC reforms as an essential part of the planned NHI depends on the active involvement and participation of PHC facility managers, ongoing training, resource availability and encouraging a culture of learning from both mistakes and successes.
